# Evidence for Local Antibiotics in the Prevention of Infection in Orthopaedic Trauma

**DOI:** 10.3390/jcm11247461

**Published:** 2022-12-16

**Authors:** Michael J. Flores, Kelsey E. Brown, Saam Morshed, David W. Shearer

**Affiliations:** Institute for Global Orthopaedics and Traumatology, Department of Orthopaedic Surgery, University of California, San Francisco, CA 94110, USA

**Keywords:** fracture-related infection (FRI), orthopaedic trauma, antibiotics, orthopedics fracture healing, bony callus, bone infection

## Abstract

Prevention of fracture-related infection (FRI) remains a substantial challenge in orthopaedic trauma care. There is evolving evidence to support the use of local antibiotics for both the prevention and treatment of musculoskeletal infection. Local antibiotics can achieve higher local tissue concentrations with a lower risk of systemic complications compared to intravenously administered antibiotics. These antibiotics may be administered in powder or liquid form without carrier, or if sustained release is desired, using a carrier. Polymethylmethacrylate (PMMA), ceramics, and hydrogels are examples of antibiotic carriers. Unlike PMMA, ceramics and hydrogels have the advantage of not requiring a second surgery for removal. The VANCO trial supported the use of powdered vancomycin in high-risk fracture cases for the reduction of Gram-positive infections; although, data is limited. Future studies will evaluate the use of aminoglycoside antibiotics to address Gram-negative infection prevention. While theoretical concerns exist with the use of local antibiotics, available studies suggest local antibiotics are safe with a low-risk of adverse effects.

## 1. Introduction

Injury accounts for over 10% of the global disease burden, with trauma accounting for the majority of injury-related disability worldwide [1]. Additionally, it has been found that the global incidence of fractures and fracture-related disability have increased substantially in recent years [2]. Since high-energy mechanisms often accompany traumatic injury, they are particularly prone to infection due to high rates of open injuries with soft tissue damage and the necessity for operative management. Even after operative debridement, infection rates have been cited anywhere between 4–63% [3,4,5]. Although less common, closed injuries have also been associated with infection [6]. Surgical site infections (SSIs) can be particularly difficult to treat, since some of the common bacterial strains causing SSI have the ability to create a protective biofilm that shields them from host immunity and systemic antibiotics [6]. These infections can lead to devastating morbidity, including delayed wound healing, fracture nonunion, amputation, and even death [7,8].

Early administration of intravenous antibiotics and thorough debridement remain the gold standard interventions to reduce infection after open fracture [9,10]. Although prophylactic systemic antibiotics have been shown to reduce infection rates, high rates of infection persist despite their use [3,11,12]. In an effort to further reduce the risk of infection, the introduction of local antibiotics into the wound after appropriate irrigation and debridement has been implemented with promising results [11,13,14,15,16,17]. This narrative review outlines the evolving evidence related to the use of local antibiotics in the prevention of infection in the setting of orthopaedic trauma.

## 2. Methods

Evidence for the current narrative review was based on articles obtained through a MEDLINE search (September 2021) using the key words “Surgical Wound Infection” (MeSH term), “Antibiotic Prophylaxis” (MeSH term), and “Local antibiotic” (text word). As the current study was not a systematic review, recommended articles and viewpoints from experts in the field of orthopaedic trauma were also included.

## 3. Rationale

The use of local antibiotics during surgical procedures has been ongoing for over a century, stemming from the pioneering work of Joseph Lister [6]. Despite their early introduction in the setting of established infections, systemic antibiotics have become the gold standard for prophylaxis as well as therapeutic antibiotics, with much less written about the use of local antibiotics [6,9,10]. However, renewed attention has been placed on the role of prophylactic local antibiotics in recent years, as high rates of surgical site infections (SSIs) have persisted [7,8].

Local antibiotics have potential advantages in the setting of traumatic injury for a variety of reasons. Vascular injury, local tissue damage, or periosteal stripping may all limit the delivery of intravenous antibiotics to the injury site [11]. In these instances, locally administered antibiotics can be more effective in achieving high concentrations at the site of injury and contamination. Additionally, local antibiotics can be administered at much higher local concentrations with lower systemic levels, decreasing the risk of complications such as nephrotoxicity or ototoxicity [3,6]. These high local concentrations of antibiotics have recently been shown not only to reduce the risk of infection, but also decrease biofilm creation and bacterial resistance [8,11,18]. Finally, the use of local antibiotics in conjunction with systemic antibiotics may result in a synergistic effect to further decrease the risk of SSI [19].

The pharmacokinetics of local antibiotics without a carrier are less studied than antibiotics administered intravenously or with a carrier. Studies of locally-applied vancomycin in spine surgery in both adults and children have shown antibiotic levels 1000-fold higher than MICs for common pathogens that persist for 2–3 days post-operatively with relatively low systemic concentrations [20,21]. However, the pharmacokinetics in trauma populations and the potential impact of impurities in local antibiotics are not well understood. 

## 4. Carriers

Local antibiotics can be delivered in multiple ways including with or without a carrier. A recent review published by the Fracture-Related Infection (FRI) consensus group summarises these strategies [22]. Additionally, an overview of the different carriers discussed in the current review can be found in Table 1. Local antibiotic carriers can be both absorbable and non-absorbable. Common carriers include polymethyl methacrylate (PMMA), biodegradable ceramics such as calcium sulfate (CaSO_4_), and hydrogels. Carriers provide a means for burst, tapered, and sustained release of antibiotics [22,23]. However, there is some concern for increased risk of the development of antibiotic resistance with carrier use due to sustained release at subtherapeutic levels [24]. Antibiotic resistance is critical to consider when using antibiotic drugs and carriers, and they should be properly assessed for safety and efficacy through high-level evidence studies and proper regulation prior to their general recommendation and use. Additionally, variations in carrier type and formulation, as well as the antibiotics used, can alter antibiotic elution and dilution profiles and the mechanical properties of their carriers [22]. It should be noted that although PMMA and other carriers are commonly used with local antibiotics, this is not an FDA approved use of these products, and hence this off-label use and associated risks should be discussed with patients prior to their application. It is also noteworthy that due to cost, most carriers are utilised for the treatment rather than prevention of infection.

**Figure 1:**
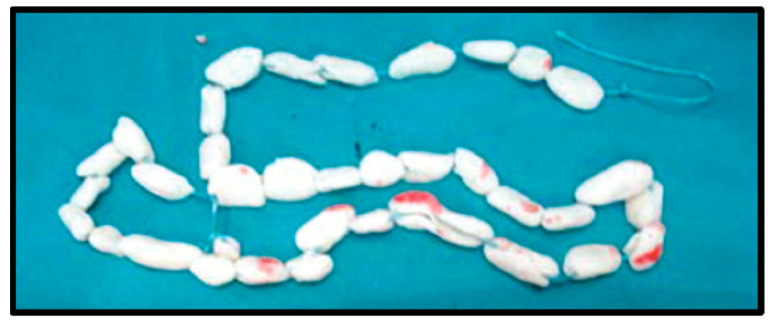
PMMA beads can be created by a hand over suture or wire, with or without the use of prefabricated templates. Beads provide no structural integrity but effectively occupy dead space and provide a higher level of antibiotic elution due to greater surface area than mono-block creations.

**Figure 2:**
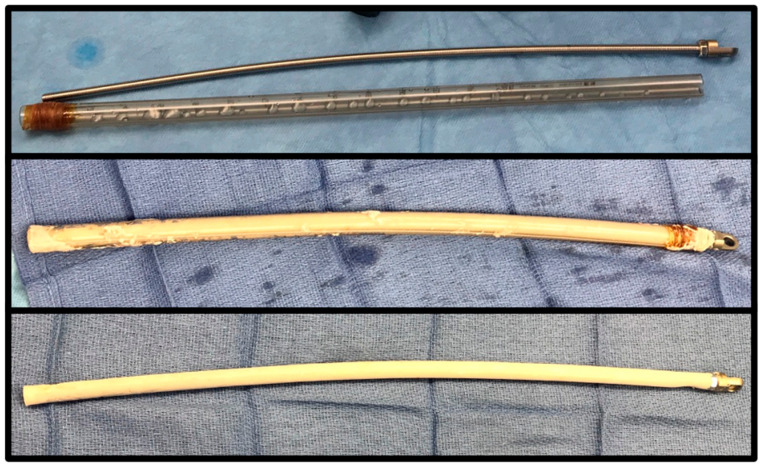
A PMMA-coated intramedullary nail can be fabricated using a chest tube or a threaded rod or guidewire. After mixing, the cement is injected into the chest tube and the rod is inserted. The tubing is then removed after the PMMA has hardened using a scalpel.

**Figure 3:**
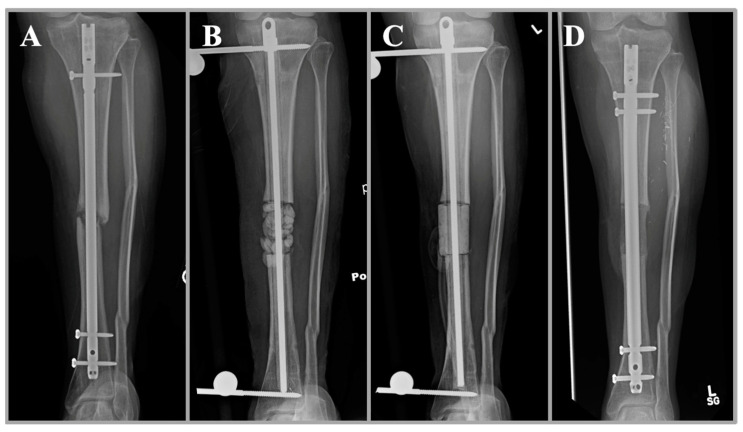
A clinical case of a 27-year-old woman with infected nonunion of the tibia after open fracture (**A**). She underwent initial irrigation and debridement with removal of hardware and placement of an antibiotic nail, antibiotic beads, and external fixation (**B**). The beads are ideal in this scenario for maximal surface area and antibiotic elution. At the same time, the antibiotic nail provides stability and local antibiotic delivery in the intramedullary space. A repeat debridement was performed with the placement of a mono-block spacer in the subsequent procedure (**C**). The mono-block spacer has the advantage of inducing a membrane for subsequent bone grafting and improved structural integrity. Healing of the fracture is shown after bone grafting with the placement of a new intramedullary nail (**D**).

**Figure 4:**
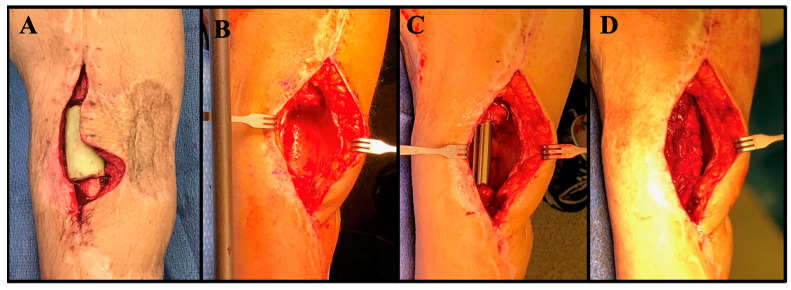
Clinical images of the same patient are shown at the time of antibiotic spacer placement (**A**), spacer removal (**B**), nail placement (**C**), and bone grafting (**D**).

PMMA is the oldest and most common carrier available. Originally designed as a bone cement, it was quickly adopted as an antibiotic carrier in the 1970’s, especially for the treatment of osteomyelitis due its ability to occupy large amounts of dead space [23,25]. PMMA is a solid rigid polymer that can be formed into beads or a mono-block spacer. During the curing phase, antibiotics can be incorporated into the polymerized matrix [22,23,26] (Figure 1). Additionally, antibiotic-infused PMMA has been used to coat orthopaedic implants [27,28] (Figure 2, Figure 3 and Figure 4). The polymer porosity, antibiotic thermal stability, antibiotic class, and use of fillers all contribute to the final PMMA-mediated antibiotic strength and timing of antibiotic release [22,29,30,31]. It is important to recognise that the addition of antibiotics to PMMA leads to a non-linear decrease in strength, which may be relevant if a load-bearing spacer is desired. Although many antibiotics have been used with PMMA, vancomycin, tobramycin, and gentamicin are the most commonly used, and when in combination, have been shown to provide a synergistic effect [22,23]. Many studies have shown that local PMMA-mediated antibiotics decrease infection risk and biofilm formation [16,23,32]. However, because PMMA is not absorbable, it typically requires a second surgical procedure for removal. In addition, although its ability to occupy dead space can be an advantage, in cases without bone loss, there may not be sufficient space to allow for the placement of PMMA. For these reasons, PMMA is more commonly used either as prophylaxis in high-energy open fracture situations with bone or soft-tissue loss or in the treatment of infection in the setting of osteomyelitis and fracture-related infection. 

Ceramics have been shown to have similar outcomes to PMMA, with the primary advantage of being absorbable and hence not requiring a secondary surgery for removal [33,34]. Common ceramics are derived from formulations of calcium sulfate or calcium phosphate. Although calcium sulfate resorbs at a much faster rate, the antibiotic elution profiles of both ceramics are similar [22,35]. However, resorption time should be taken into account when using ceramics, as slower resorption times can be useful in persistent infections, but can potentially be a nidus for future infection [35]. Although their use in local antibiotic prophylaxis is relatively new [23], they have been shown to maintain antibiotic concentrations longer than PMMA, leading to decreased infection and biofilm formation [34,36]. Like PMMA, ceramics have been proven effective as an antibiotic carrier in other situations such as in treating osteomyelitis [33,37,38].

Hydrogels are emerging as an alternative antibiotic carrier with many potential advantages, but there have been less clinical studies investigating their efficacy [22]. Hydrogels are a polymer like PMMA but have the advantage of being absorbable like ceramics [22]. Additionally, they have been shown to not interfere with bone healing [39]. Hydrogels have a shorter release period due to their rapid resorption and lack the same structural integrity as other carriers due to their gel-like consistency [22]. This makes them better suited for prophylactic situations where it is less likely for dead space to be present and longer antibiotic elution periods are not needed. Since hydrogels are highly hydrophilic with a unique scaffold, they can be prepared for various situations and adapt or react to various environmental changes [40]. This makes them especially useful for targeted effects.

**Table 1:** Common Antibiotic Carrier Characteristics.

Carrier	Materials	Antibiotics	Benefits	Disadvantages	References
PMMA	Polymethylmethacrylate	Heat stable only (Aminoglycosides, Glycopeptides, Tetracyclines, and Quinolones)	Availability, Occupies dead space	Not absorbable, long elution profile	[22,23,26]
Ceramics	Calcium sulfate, calcium phosphate, or a combination	Aminoglycosides Glycopeptides Lipopeptides	Absorbable, faster resorption and elution profiles	Possible toxicity or hypercalcemia (rare), wound drainage, cost	[22,34,35]
Hydrogels	PCLA-PEG-PCLA tri-block, poly(ether ester) SynBiosys, etc.	Variable	Absorbable, fast resorption, variety	Shorter release period, lack structural integrity, cost	[22,39,40]

So-called “naked” local antibiotics, including aqueous or powder formulations, deliver antibiotics without a carrier. Aqueous formulations are one of the earliest described forms of local antibiotics in the literature and are injected into the wound after wound closure; whereas, powdered formulations are placed into the wound prior to closure. These methods are advantageous in that they cost less than other delivery methods; however, their effect is shorter lasting [22]. For example, local vancomycin powder is cited in literature to range between $2.50/g to $44/g (€3.22/g to €56.72/g using the average 2017 exchange rate of 1.289 USD) [41,42]. In contrast, one cost-effective analysis from the Netherlands showed the mean cost of PMMA per patient with osteomyelitis to be €365.00, while the use of a sulfur-based ceramic glass was €2007.20 [43]. Another economic analysis in Italy documented the price of PMMA for prosthetic implants to be €480, while an antibiotic hydrogel costs €1170 [44]. 

There are multiple variables to consider in deciding which antibiotic to pair with the different carriers. It is important to choose an antibiotic that acts against the pathogen(s) identified in the wound culture or can effectively act against a wide range of pathogens. Additionally, care must be taken to ensure the antibiotic has a proper toxicity and hypersensitivity profile at the site and a low rate of resistance from the pathogen of interest [29]. In the case of PMMA, which uses an exothermic reaction to create the polymer, thermal stability of the antibiotic is also necessary. It has been shown that beta-lactam antibiotics are not heat stable and should not be used with PMMA, while common heat stable antibiotics include aminoglycosides, glycopeptides, tetracyclines, and quinolones [22,29]. Although certain ceramic formulations also go through an exothermic reaction during their synthesis, the materials and processes vary. Currently, the antibiotics used in ceramic carriers include aminoglycosides, glycopeptides, and lipopeptides [35]. In contrast, since hydrogels are a water-soluble polymer with various preparations, many different antimicrobial substances have been incorporated into hydrogel polymers including metal nanoparticles and antibiotics [40].

## 5. Effectiveness in Trauma

There is evolving evidence regarding the general efficacy of prophylactic local antibiotic administration. While there are many observational studies that validate the efficacy of local antibiotics in the prevention of SSI in high-risk fractures, there is only one published prospective randomised controlled clinical trial (RCT) to evaluate their efficacy [45].

The Local Antibiotic Therapy to Reduce Infection After Operative Treatment of Fractures at High Risk of Infection (VANCO) trial evaluated the efficacy of local vancomycin in preventing surgical site infections after fracture surgery [11]. This multi-centre clinical trial collected data from 34 US trauma centres who participated in the Major Extremity Research Consortium (METRC). The trial included participants aged 18–80 years with closed or open tibial plateau and pilon fractures requiring staged treatment and randomised them to either receive standard of care or the placement of 1 gram of vancomycin locally prior to wound closure. The time-to-event estimates for surgical site deep infection rates at 6 months follow up were 6.4% in the group who received local vancomycin and 9.8% in the control group (*p* = 0.06). In a post-hoc subgroup analysis, the rate of a Gram-positive infection was reduced from 6.8% to 3.3%, (*p* = 0.02) while a Gram-negative infection was equal between the two groups. It is important to note that significance in the VANCO trial was only achieved in this subgroup analysis. Additionally, this subgroup analysis was not pre-specified and hence should be interpreted with caution. However, the findings of this single level 1 trial generally support the use of vancomycin in the reduction of Gram-positive SSI in high-risk fractures. 

As a follow up to the VANCO trial, the METRC group is beginning to enrol a new multicentre trial, the Topical Antibiotic Therapy to Reduce Infection After Operative Treatment of Fractures at High Risk of Infection (TOBRA) study. The TOBRA trial will compare the efficacy of combining local vancomycin and tobramycin to local vancomycin alone in the prevention of deep SSI in high-risk fractures [46]. The target enrolment for the study is 1900 participants, and results are anticipated to be available in May 2024 [46].

Lawing et al. described the use of a local gentamicin injection in open fractures to prevent infection and reported their results in an observational study before and after implementation of its use [3]. The study found that the deep and superficial infection rate was reduced from 19.7% to 9.5% (*p* = 0.010). Although the study was not randomised, after adjustment using a regression model, local antibiotics were an independent predictor of lower infection risk [3].

The Local Gentamicin for Open Tibial Fractures in Tanzania (GO-Tibia) trial is an ongoing RCT being conducted at the Muhimbili Orthopaedic Institute in Dar as Salaam, Tanzania in collaboration with the Institute for Global Orthopaedics and Traumatology (IGOT) at the University of California, San Francisco [47]. The GO Tibia Trial is aimed to evaluate the efficacy of locally-injected gentamicin on the risk of fracture-related infection (FRI) after open tibia fractures [47]. In contrast to the VANCO and TOBRA trials, the GO Tibia study is masked with a saline placebo control. A pilot trial was recently completed (publication pending), and enrolment in a larger definitive trial is scheduled to begin in January 2022.

## 6. Potential Limitations

Although prophylactic antibiotics may reduce the risk of infection [3,11,15,16,48,49], some studies have demonstrated inconclusive results [50,51]. Furthermore, as demonstrated in this review, there are currently few high-level evidence studies on prophylactic antibiotic use in musculoskeletal trauma. In spine literature, there seems to be little added benefit of local vancomycin in low-risk cases where infection rates are already low [50]. Furthermore, high antibiotic concentrations may be toxic to local tissues. One in-vitro study showed that local antibiotics may have osteocyte toxicity [52], but animal studies have not shown an effect on bone healing [53]. Additionally, studies in humans have not shown an association between local antibiotics and nonunion or delayed healing. Another potential limitation to local antibiotic use is the development of antibiotic resistance with prophylactic usage, particularly with second-line antibiotics such as vancomycin. However, local use results in low systemic levels for short durations, hence local use would be anticipated to have lower risk of developing resistance than systemic antibiotics. One theoretical concern with local vancomycin is that impurities in the formulation may act as a nidus for the development of future infections. Another concern is the selection of bacteria, such as Gram-negative organisms, with local vancomycin. However, a meta-analysis on this topic has shown no evidence of selection of pathogens [17]. The TOBRA study will further address this issue by broadening coverage. Finally, the use of prophylactic local antibiotics is an off-label use, and as such, may cause unforeseen adverse events. Additionally, this unregulated use of local antibiotic prophylaxis leads to a lack of quality control, standardisation, and limited understanding of elution and diffusion characteristics. Although there has been concern for the occurrence of adverse events such as nephrotoxicity, ototoxicity, and hypersensitivity reactions with the use of local antibiotics [54,55], there has been little evidence of these complications [19,56,57]. However, given that the potential limitations and complications of prophylactic local antibiotic use are currently addressed by lower-level evidence studies, the use of prophylactic local antibiotics should be considered with caution until further evidence from randomised controlled trials and regulatory bodies have been obtained.

## 7. Conclusions

There is evolving evidence supporting the use of prophylactic local antibiotics in the prevention and treatment of infections in orthopaedic trauma. Many studies show promising results of their efficacy with few, if any, reported adverse effects despite theoretical concerns. However, current high-level research is limited, and further well-designed randomised controlled trials are needed before definitive recommendations can be made in regard to their use. Greater awareness and appropriate use of local antibiotics has the potential to reduce the global impact of fracture-related infection.
